# Cardiopulmonary Resuscitation and Defibrillator Use in Sports

**DOI:** 10.3389/fcvm.2022.819609

**Published:** 2022-02-15

**Authors:** Mafalda Carrington, Rui Providência, C. Anwar A. Chahal, Flavio D'Ascenzi, Alberto Cipriani, Fabrizio Ricci, Mohammed Y. Khanji

**Affiliations:** ^1^Department of Cardiology, Hospital do Espírito Santo de Évora, Évora, Portugal; ^2^Department of Cardiology, Barts Heart Centre, Barts Health NHS Trust, London, United Kingdom; ^3^Department of Cardiology, Newham University Hospital, Barts Health NHS Trust, London, United Kingdom; ^4^Institute of Health Informatics Research, University College London, London, United Kingdom; ^5^Cardiovascular Division, University of Pennsylvania, Philadelphia, PA, United States; ^6^Department of Cardiovascular Medicine, Mayo Clinic, Rochester, MN, United States; ^7^Centre for Inherited Cardiovascular Diseases, WellSpan Cardiology, Lancaster, PA, United States; ^8^Division of Cardiology, Department of Medical Biotechnologies, University of Siena, Siena, Italy; ^9^Department of Cardio-Thoraco-Vascular Sciences and Public Health, University of Padua, Padua, Italy; ^10^Department of Neuroscience, Imaging and Clinical Sciences, “G.d'Annunzio” University of Chieti-Pescara, Chieti, Italy; ^11^Department of Cardiology, Casa di Cura Villa Serena, Città Sant'Angelo, Italy; ^12^Department of Clinical Sciences, Lund University, Malmö, Sweden; ^13^NIHR Biomedical Research Unit, William Harvey Research Institute, Queen Mary University, London, United Kingdom

**Keywords:** cardiopulmonary resuscitation, automated electrical defibrillator, implantable cardioverter defibrillator, sudden cardiac death, cardiac arrest, out of hospital cardiac arrest, athlete, sports cardiology

## Abstract

Sudden cardiac arrest (SCA) in young athletes is rare, with an estimated incidence ranging from 0.1 to 2 per 100,000 per athlete year. The creation of SCA registries can help provide accurate data regarding incidence, treatment, and outcomes and help implement primary or secondary prevention strategies that could change the course of these events. Early cardiopulmonary resuscitation (CPR) and defibrillation are the most important determinants of survival and neurological prognosis in individuals who suffer from SCA. Compared with the general population, individuals with clinically silent cardiac disease who practice regular physical exercise are at increased risk of SCA events. While the implementation of national preparticipation screening has been largely debated, with no current consensus, the number of athletes who will be diagnosed with cardiac disease and have an indication for implantable defibrillator cardioverter defibrillator (ICD) is unknown. Many victims of SCA do not have a previous cardiac diagnosis. Therefore, the appropriate use and availability of automated external defibrillators (AEDs) in public spaces is the crucial part of the integrated response to prevent these fatalities both for participating athletes and for spectators. Governments and sports institutions should invest and educate members of the public, security, and healthcare professionals in immediate initiation of CPR and early AED use. Smartphone apps could play an integral part to allow bystanders to alert the emergency services and CPR trained responders and locate and utilize the nearest AED to positively influence the outcomes by strengthening the chain of survival. This review aims to summarize the available evidence on sudden cardiac death prevention among young athletes and to provide some guidance on strategies that can be implemented by governments and on the novel tools that can help save these lives.

## Introduction

The annual incidence of out-of-hospital cardiac arrest (OHCA) in the general population is estimated between 67 and 170 per 100,000 inhabitants in Europe (1) and 57 per 100,000 inhabitants in the United States (US) (2), widely varying between and within countries. In addition, the causes of sudden cardiac death (SCD) might also differ among different countries, possibly because of differences in population genetics and myocardial substrate and the systematic preparticipation evaluation of athletes (3). Cardiopulmonary resuscitation (CPR) initiated by bystanders is reported to be performed in about half of cases, with significant differences between countries (4).

**Figure 1:**
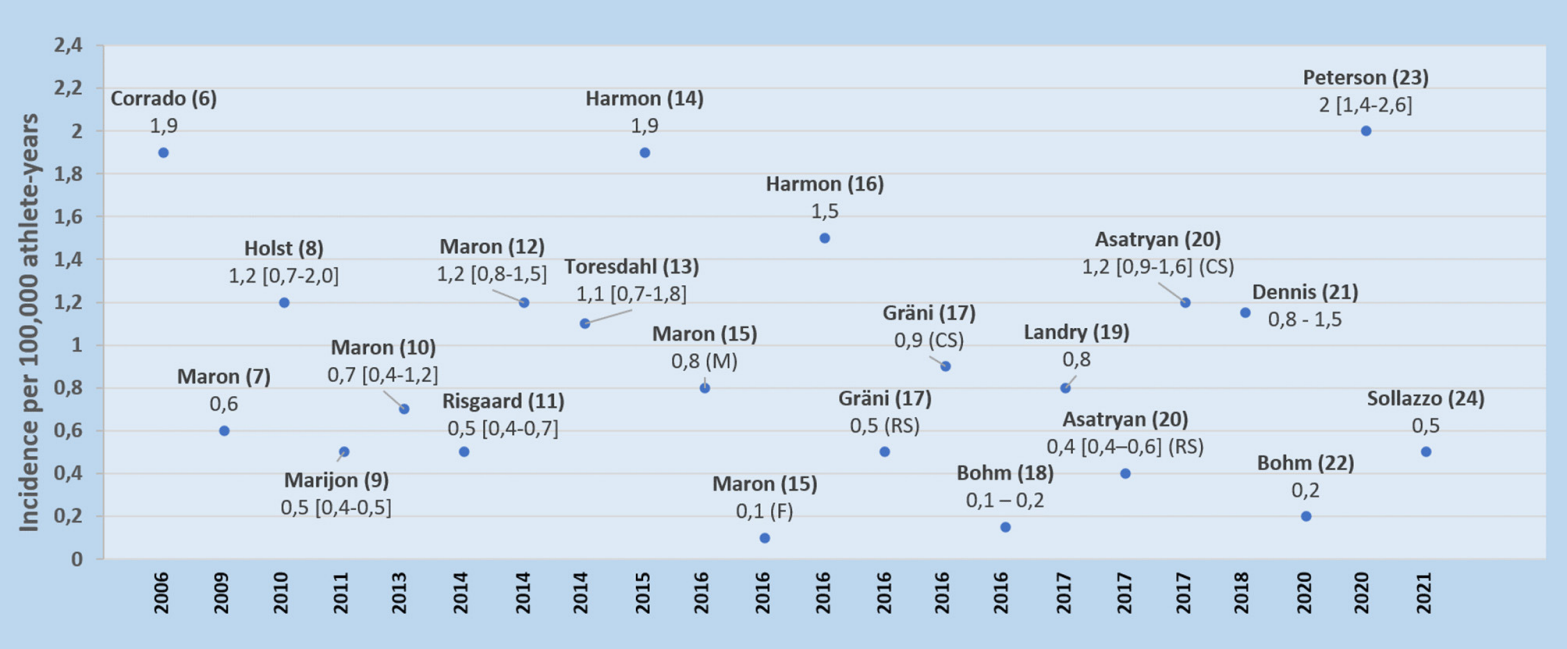
Representation of incidences of sports-related sudden cardiac arrests by ascending date of publication. Mean incidence [95% CI included where available]. CS, competitive sports; F, female; M, male; RS, recreational sports.

Sudden cardiac arrest (SCA) or SCD in young athletes is even rarer, although it is often an event of great public attention. In 2014, *Harmon* and colleagues reviewed the incidence of SCD in athletes and concluded that studies with higher methodological quality consistently yielded incidence rates in the range of 1:40,000–1:80,000, and assumed an overall incidence of 1:50,000 in young athletes is a reasonable estimate (5). In Table 1 and Figure 1, we summarize data from studies published from 2006 to 2021, with incidences of SCA ranging from 0.1 to 2 per 100,000 athlete-year (6–24). Most of these studies have shown that the majority of SCA events occur during exercise, despite possible selection bias because of the study sources including databases of more commonly sports organization and media report reviews.

**Table 1:** Incidence of sports-related sudden cardiac arrests.

**Reference**	**N**°**of cases**	**Country/Population**	**Years**	**Incidence (athlete-years)**	**Methods**	**Context of events**	**Age range (mean)**
Corrado et al. (6)	55	Venetto, Italy	1979–2004	1.9/100,000	Prospective study, period, including clinical pathological review, regional newspaper screening and postmortem examination to ascertain the causes of SD of screened athletic population.	80% during sports activity, 11% immediately afterward	12–35
Maron et al. (7)	1,049	USA	1980–2006	0.6/100,000	Prospective US National Registry of Sudden Death in Athletes	80% during of immediately after physical exertion, 20% unassociated with physical activity	8–39
Holst et al. (8)	15	Danish young population (5.4 million)	2000–2006	1.2/100,000	Nationwide retrospective study, all death certificates reviewed by 2 independent physicians for possible sports-related SCD	33% while running and 33% while playing soccer. 73% occurred in sports arena.	12–35
Marijon et al. (9)	820	France	2005–2010	0.5/100,000	Prospective surveillance of: (1). National ambulance service reporting, (2). Web-based screening of media releases	6% in young (10–35 years-old) competitive athletes	10–75 (40)
Maron et al. (10)	13	Minnesota State High Schools, USA	1986-2011	0.7/100,000	Prospective US National Registry of Sudden Death in Athletes	54% during competition and 46% during practice or training	12-18
Risgaard et al. (11)	44	Danish young population	2007–2009	0.5/100,000	Nationwide retrospective study, all death certificates reviewed by 2 independent physicians for possible sports-related SCD	75% occurred during non-competitive sports activities. 39% while running and 30% while cycling. 68% occurred in public arena.	12–49
Maron et al. (12)	64	College Athletes, USA	2002–2011	1.2/100,000	Prospective US National Registry of Sudden Death in Athletes and the National Collegiate Athletic Association database	9% during competition, 36% during practice, 22% during recreational activity, 33% unassociated to physical activity	17–26
Toresdahl et al. (13)	18	High school students, USA	2009-2011	1.1/100,000	Prospective observational study of 2149 US high schools participating in the National Registry for AED Use in Sports	100% associated with physical activity	High-school years
Harmon et al. (14)	79	USA	2003–2013	1.9/100,000	Prospective surveillance of: (1). NCAA Resolutions List, (2). Parent Heart Watch databas, (3). NCAA insurance claims	56% during exertion, 22% at rest, 14% during sleep	17–24
Maron et al. (15)	842	USA	1980–2011	0.8/100,000 in males 0.1/100,000 in females	Prospective US National Registry of Sudden Death in Athletes participating in competitive athletics who had an autopsy-confirmed cardiovascular diagnoses	26% during competition, 39% during practice, 17% during recreational activity, 17% unassociated to physical activity	15–24
Harmon et al. (16)	104	Seven states of the USA	2007–2013	1.5/100,000	Parent Heart Watch database, based on prospective systematic searches of media reports and queries	80% during exertion	14–18
Gräni et al. (17)	69	German and French-speaking regions of Switzerland (7.0 million)	1999–2010	0.5/100,000 in recreational sports 0.9/100,000 in competitive sports	Retrospective review all forensic reports	Incidences refer to whether each type of sports were performed within the 24-h preceding the SCD	10–39
Bohm et al. (18)	144	Germany	2012–2014	0.1–0.2/100,000	Prospective surveillance of: (1). Web-based platform to record sports-related SCD and SCA cases in competitive and recreational athletes, (2). Media-monitoring, (3). Cooperation with 15 institutes of forensic medicine	26% survived. 85% during sports activity, 15% up to 1 hour after sports cessation.75% in public sports facilities	10–79 (47)
Landry et al. (19)	74	Specific area of Ontario, Canada (6.6 million)	2009–2014	0.8/100,000	Retrospective study, review of the Rescu Epistry cardiac arrest database to identify all out-of-hospital cardiac arrests that occurred during participation in a sport	74% during non-competitive sports	12–45
Asatryan et al. (20)	52	German-speaking regions of Switzerland (5.6 million)	1999–2010	0.4/100,000 in recreational sports 1.2/100,000 in competitive sports	Retrospective review all forensic reports	Incidences refer to whether each type of sports were performed within the 24-h preceding the SCD	10–39
Dennis et al. (21)	216	New South Wales, Australia	2006–2015	0.8-1.5/100,000	Retrospective study, review of the database of the department of forensic medicine to identify all sudden deaths related to sports	48% during organized sports, 19% during regular sports and 31% leisure sports activity	7–65
Bohm et al. (22)	349	Germany	May 2012 –April 2018	0.2/100,000	Prospective surveillance of: (1). Web-based platform to record sports-related SCD and SCA cases in competitive and recreational athletes, (2). Media-monitoring, (3). Cooperation with the German Resuscitation Registry, (4). Cooperation with 15 institutes of forensic medicine.	31% survived. 82% during sports activity, 11% within 30 min and 5% between 30 min-1hafter sports cessation.	10–79 (48)
Peterson et al. (23)	331	USA	2014–2018	2/100,000	Prospective surveillance of: (1). Traditional and social media sources, (2). Reportings to National Center for Catastrophic Sports Injury Research and University of Washington Medicine Center for Sports Cardiology, (3). Regular review of student-athlete deaths on NCAA Resolutions List, National Federation of State High School Associations and Parent Heart Watch database	74% during exercise, 4.2% within 1h after exercise, 12.1% at rest, 6.0% during sleep.	11–29 (17)
Sollazzo et al. (24)	98	Italy	2019	0.5/100,000	One year- long Google search was performed using mandatory and non-mandatory keywords	51% during sports practice, 9% immediately afterwards, 14% during sleep, 25% at rest or during day-to-day activities	12% under 18–years-old, 27% between 18 and 35, 61% over 35

*AED, automated external defibrillator; NCAA, National Collegiate Athletic Association; SCD, sudden cardiac death; USA, United States of America*.

The wide variation in incidences reported might reflect the underreporting of SCA events and the lack of appropriate national sports registries (25) that might allow a more precise epidemiological description of the problem.

This document aims at summarizing the available evidence on SCD prevention among young athletes, and to provide some guidance on strategies that can be implemented by governments and on the novel tools that can help save these lives.

## Increasing Training and Delivery of Bystander CPR

Governments and sports institutions could invest in educating members of the public, security personnel, and healthcare professionals in the identification of SCA, calling for appropriate help, early initiation of effective CPR, and automated external defibrillator (AED) use, which can be highly lifesaving (26, 27). People are generally unaware of how to deal with SCA events, although teaching CPR maneuvers is valuable and easy, as evidenced that even training as short as 2 h can lead to a major increase in the willingness to start CPR and AED use (28). In addition, after the Australian government provided basic life support (BLS) and AED use training, a 6-month follow-up survey demonstrated highly accurate answers to clinical scenarios involving AED use, although only half of the respondents reported having access to an AED (29).

Training and raising awareness among the population to CPR and eventually AED use should be provided as part of the school civic education, as it is an important issue of public health. This has already been recognized by governments of many countries across Europe and in the US that have legal requirements for CPR education in schools (30, 31). However, it is not known whether legislation has translated into implementation, as demonstrated by a Danish group that performed a nationwide study and demonstrated that school CPR training has not been successfully implemented following 8 years of mandating legislation (32). Over 10 years, temporal trends in volunteer CPR delivery and long-term survival were studied in Denmark, after several national initiatives were implemented to strengthen bystander resuscitation attempts (33). These initiatives included mandatory CPR training in elementary schools, as well as when acquiring a driving license, combined with an increase in voluntary first-aid training (33). An increase in bystander CPR was verified, and it was significantly associated with a concomitant increase in survival following OHCA (33). In 2015, the WHO endorsed the European Resuscitation Council initiative “Kids save lives.” This initiative is meant to deal with the gap in the education of CPR, starting with training children from the age of 12 years, for 2-h every year, as a part of educational project of the schools (30). However, in 2018, *Semeraro* et al. found that although education of children in resuscitation is mandatory by law in schools in six countries in Europe and it is a recommendation in another 24 countries, full implementation has not yet been achieved in the majority of them (34). In the US, the effectiveness of school-based AED programs was also studied and it was high, with an AED application in 85% of SCA victims and 85% survival to hospital discharge among students after an event (35).

## Effectiveness of Bystander Defibrillation

### Efficacy of CPR and AED Use

Sudden cardiac arrest may be caused by asystole, complete heart block with ventricular standstill, electromechanical dissociation/pulseless electrical activity (PEA), pulseless ventricular tachycardia (VT), or ventricular fibrillation (VF). While patients who present with asystole or pulseless electrical activity have a poor prognosis despite CPR delivery, those in whom the first documented rhythm is VT or VF can be effectively treated by defibrillation. In a prospective national survey of the national French ambulance service, involving subjects with 10 to 75 years who suffered sports-related SCD, the first reported rhythm was VF or pulseless VT in 47%, asystole in 42%, and PEA in 11% (9). Therefore, a relevant proportion of underlying arrhythmias for sports-related SCA can potentially be reverted by an AED shock (36).

Although primary prevention by screening professional athletes for cardiovascular diseases at risk for SCA is undertaken, combining this with increased CPR training and the availability of AED, will increase the likelihood of survival of individuals with unpredictable SCA (37). The important role of bystander-provided defibrillation in individuals who suffer OHCA is corroborated by several studies. After the implementation of a nationwide CPR and AED use training in school students in Japan, a retrospective study in elementary and middle school students demonstrated that children were more likely to be defibrillated by bystanders and had better neurological outcomes and 1-month survival when the cardiac arrest occurred in schools and other public places (38). Similarly, a systematic review of SCA in schools has shown that outcomes are better than when occurring at other locations, probably because of more frequent witnessed collapses and bystander CPR delivery (39). Aside from schools, other public places such as airports, that serve millions of passengers each year, where the risk of occurrence of SCA events is higher, and where short response times that save lives have been evaluated. As an example, in a prospective study in the three Chicago airports, equipped with seventeen AEDs, eighteen ventricular fibrillations occurred throughout years 2 years (40). In four of these cases, defibrillators were neither nearby nor used within 5 min, and the patients died (40). The overall one-year survival rate with a good neurological outcome was 56% (40). AEDs were also proven to be useful and efficacious when used on commercial airplanes, where early response by the prehospital emergency services is usually not possible (41). In a study performed in this setting, the rate of survival to hospital discharge shock after the shock was 40% and there were no inappropriate shocks (41). Finally, in a prospective observational cohort which included 2,500 shockable OHCA observed by the public, the authors found that survival to hospital discharge [adjusted odds ratio (OR) 2.62, 95% CI 2.07–3.31] and favorable functional outcome (adjusted OR 2.73, 95% CI 2.17–3.44) were significantly higher when a bystander rather than emergency medical services (EMSs) professionals provided the initial shock, and the benefit of bystander shock increased progressively as EMS response time became longer (42). Despite the high efficacy of AED use in public places, there is limited AED availability in public spaces in the majority of countries (1) and the scenario is as abysmal in nonprofessional athletic clubs (28, 43, 44). Furthermore, bystander AED use occurs in <2% of OHCAs, and the median arrival time of EMS can extend to 30 min in remote areas, where drone-delivery AED systems might increase the chances of survival (45).

### Cost-Effectiveness of AED Use

The cost-effectiveness of training lay volunteers in CPR and AED use and public access to AED has not been well-studied (46), but may be considered a limitation of the strategy. In the Public Access Defibrillation (PAD) trial, 993 community facilities were randomly assigned to a structured emergency response strategy involving lay volunteers trained either in CPR alone or in CPR and the use of AEDs (47). Community facilities included shopping centers, office buildings, recreational/entertainment complexes, hotels, and apartment complexes that were eligible if an equivalent of at least 250 adults of more than 50 years were present for 16 h a day, or if there was a history of one SCA every 2 years (47). The mean number of volunteers trained per facility was 20 (range 1–149). These were laypersons who received training at enrollment and were retrained after 3–6 months and at least once after that (47). The addition of AED use before EMS arrival improved SCA survival to hospital discharge by 2-fold (95% CI 1.07–3.77) (47) and defibrillation by volunteers was associated with an incremental cost of mean $46,700 (95% CI $23,100–$68,600) per quality-adjusted life year, compared with CPR alone, which was stated to be an acceptable difference (48). In the CPR plus AED group, equipment plus training costs $4,453 per cardiac arrest (48). Therefore, in the athlete population, assuming an incidence of 1 per 50,000 athletes-year (5), one should expect to spend 28 times more than in the general population (estimated incidence of SCA in the US: 57 per 100,000 inhabitants—see Introduction part), thus $124,684 per 1 athlete-life saved. Although this may not be cost-effective if only considering the athlete, we should not forget about the risk of SCA in spectators, particularly those older and with cardiovascular risk factors. Furthermore, in a meta-analysis of 1,583 cases (including data from the PAD trial), the number of SCA needed to be treated (NNT) by nonhealthcare professionals trained in CPR plus AED to gain one survival to hospital admission was 17 (49).

### Availability of AEDs

Competitive athletes who collapse on exertion can theoretically be rapidly assisted by trained healthcare professionals or club personnel and be defibrillated when an AED is available. SCA was associated with an 8-times higher survival rate compared with nonsports-related SCA, mainly because of better initial management, including bystander CPR and AED use (50). Nevertheless, many studies have demonstrated suboptimal CPR and AED application (28, 43, 44). While professional athletes frequently play in competitions under the supervision of a medical team that is ready to act whenever there is a collapse on the field, amateur athletes are more vulnerable to death after SCA gave the poorer ability of bystanders to assist in this setting. In the Gaelic Athletic Association (GAA), one of the great amateur sporting associations in the world, a survey demonstrated that 60% of the respondents reported that their club owned an AED and only 53% noted to have received formal training to use it (43). Several other studies have demonstrated that the knowledge and willingness to use AED is relatively low among participants in amateur clubs (28). As an example, among 218 amateur sports clubs in Ireland, 81.3% owned an AED and 12.9% admitted to not maintaining it on a regular basis (44).

### Recent Developments in AED Technology

Recently, AED suppliers have worked on devices that are smaller, weight lightweight, and designed to be used by anyone, even children. As an example, HeartHero AED is a miniaturized, portable, and user-friendly AED that guides the user through the CPR process with auditory and visual guides (https://hearthero.com). Although it is not yet FDA approved, it is already available in 33 countries (cost ~€595, £495) with the potential to become a useful tool for people at increased risk of SCA that can store the potentially life-saving device at home or carry it with them, ensuring instant access to an AED. In addition, some new AEDs technology allows recording data from the moment it is attached to the patient and makes it transmittable to emergency services and hospitals, thus providing more accurate patient care. Potential limitations for wider use could include the cost for individuals and potential cost-effectiveness given the need for maintenance for appropriate functioning and the fact that those at increased risk of SCA may already have or be eligible for an implantable defibrillator.

## Smartphone APP To Locate Nearby AEDS and CPR Trained Laypersons

In the 2021 European Resuscitation Council guidelines, it is highly encouraged that potential first responders (layperson, police officers, firefighters, and off-duty healthcare professionals) who are near the SCA victim should be notified through an alerting system using a smartphone app or text messaging (Table 2) (30). An example of such a system has been reported by Dutch investigators, consisting of a text-message alert system activated by the EMS to dispatch lay rescuers who are close to the victim and locate nearby AEDs (51). This system implementation was associated with a connection of the patient to AED in <6 min in 12.3% and early (≤ 6 min) defibrillation in 7.3% of the cases. In addition, a Swedish group published a blinded randomized controlled trial, where a mobile phone positioning system was used to locate trained responders within 500 meters of patients suffering SCA, at the moment EMS ambulances were dispatched. This system was associated with significantly increased rates of bystander-initiated CPR (62 vs. 48%, *p* < 0.001) (52). In another study from Sweden, when testing a similar system, lay responders arrived at the scene before the EMSs in 26% of the cases, and in 9% it they was able to attach an AED (53). Heartrunner™ (Heartrunner Sweden AB, Sweden) is the Swedish app that connects the EMS with 188,500 citizen responders and 5,000,000 AEDs around Sweden and Denmark (https://heartrunner.com/about-the-system/). When comparing citizen responders arriving before EMS, the early arrival of Heartrunner™-dispatched citizen responders was associated with almost 2-fold increased odds for bystander CPR and more than 3-fold increase in odds for bystander defibrillation (54). Another big player in lay response recruiting and AED localization in the United Kingdom is the GoodSAM™ app (GoodSAM LTD, United Kingdom), which accounts for a database of more than 50,000 AEDs and over 40,000 volunteers registered worldwide (https://www.goodsamapp.org). This app also has a GoodSAM Alerter™ version (GoodSAM LTD, United Kingdom), where laypeople can not only register AEDs, but also press the “Call for Help button” when they witness an emergency or need to get help quickly, and therefore activate both the EMS and the nearest lay volunteer. Finally, many useful apps are yielding multiple exercises, knowledge quizzes, and other information about CPR and cardiac arrest and can be easily downloaded for free by lay people [e.g., Hartstichting™, Netherlands, and the Resuscitation Council UK Lifesavers game app (https://www.resus.org.uk/public-resource/how-we-save-lives/lifesaver-learning/lifesaver)].

**Table 2:** First responder notification and contactless cardiac arrest detection systems using smart devices.

**Name of the app/system**	**Launch year**	**Country of origin**	**Countries of implementation**	**Software**	**AED registration/ localization**
Heartrunner™	2010	Sweden	Sweden, Denmark	Free app available in iOS and Android stores	Yes
GoodSAM™	2013	United Kingdom	United Kingdom, Australia, US, Brazil, Ireland, Finland, Spain	Free app available in iOS, Android and Windows Phones stores	Yes
EHRA First Responder App	2017	Germany	Germany	Free app available in iOS and Android stores	No
HartslagNu CPR call system	2018	Netherlands	Netherlands	Registration site: https://hartslagnu.nl/	Yes

*App, application; CPR, cardiopulmonary resuscitation; USA, United States of America*.

## Athletes With Potentially Arrhythmogenic Diseases

### Causes of SCA in Athletes

The causes of SCA/D in young athletes vary among different series and countries due in part to the varying methods of referral and ascertainment. While some authors recognized hypertrophic cardiomyopathy (HCM) as the most frequent cause of SCD in athletes from the United States (15, 23) others identified autopsy negative sudden unexplained death (AN-SUD) as the single most common etiology (14, 55). Arrhythmogenic right ventricular cardiomyopathy has been reported to account for approximately one-fifth of fatal cases in the Veneto Region of Italy (56). Different age groups, ethnicity, and genetics distribution, and also the inclusion of cases of SCA in SCD cohorts may all account for the varying etiologies found in these studies. *Peterson* and collaborators investigated the etiology of SCA/D in US competitive athletes (mean age 16.7 (11–29) years), by reviewing autopsy reports, death certificates, and medical records (23). In this prospective study, the most common cause of SCA/D across all age levels was HCM (21%), followed by idiopathic left ventricular hypertrophy (LVH) (13%), coronary artery anomalies (12%), AN-SUD (10%), arrhythmogenic cardiomyopathy (6%), long QT syndrome (5%) and *commotion cordis* (5%) (23). Similarly, *Maron* and collaborators found that HCM (36%) was the single most common cause of SCD in young athletes (mean age 19 ± 6 years), followed by coronary artery anomalies (19%) and idiopathic LVH (9%) (15). This contrasts with finding from another US study in which AN-SUD was the most frequent etiology of SCD, found in 25% among athletes 17–24 years of age (14). It was followed by coronary artery anomalies (11%), myocarditis (10%), and coronary artery disease (10%), and the incidence of cases of HCM and idiopathic LVH were even lower (8% each) (14). In a United Kingdom registry including athletes aged 29 ± 11 years (range: 7–67 years), AN-SUD (42%) was the most common cause across all age levels, followed by idiopathic LVH or fibrosis (16%) and arrhythmogenic right ventricular cardiomyopathy (13%), HCM and coronary artery anomalies accounting for only 6 and 5%, respectively (55).

### Sports Practice and Borderline Indications for an ICD Implantation in Athletes

Implantable cardioverter defibrillator (ICD) indications in athletes should not be different from those in the general population (57, 58). Also, the desire of the athlete to continue sports competition should not represent the primary indication for ICD implantations (58–60), an option that may seem particularly appealing in some patients with cardiomyopathies and channelopathies in whom exertion may increase the risk of arrhythmias. As an example, in patients with asymptomatic long QT syndrome (LQTS) without a prolonged QTc interval (genotype-positive/phenotype-negative), an ICD should only be considered if clinically indicated, namely, if the patient develops symptoms such as palpitations or syncope despite treatment with beta-blockers (57).

In these patients with LQTS, sports participation can be considered, depending on the type and setting of sports, type of genetic mutation, and symptoms (60). Although recent 2020 European guidelines (60) still restrict all the phenotype-positive athletes with LQTS from competitive sports, there is data to support return-to-play approval when patients are optimally treated and have preventive measures and annual follow-up appropriately implemented (61). This study included 494 athletes with LQTS who were given return-to-play approval by a single genetic cardiologist, 16% of whom were symptomatic before diagnosis, and 12% of whom had an ICD. Over a combined follow-up of 2 years, there was no LQTS-sports associated mortality and only 6% had one or more nonlethal LQTS-associated cardiac events.

Recommendations regarding sports participation in patients with channelopathies and arrhythmogenic cardiomyopathies may differ depending on geography. For instance, the 2015 American Heart Association/American College of Cardiology (AHA/ACC) guidelines allowed competitive sports participation in patients with symptomatic or electrocardiographic evident channelopathies (except for swimming in previously symptomatic LQTS1 (*KCNQ1*) mutation carriers), as long as appropriate precautionary measures (e.g., available AED) and disease-specific treatments are in place, and the athlete has been asymptomatic on treatment for at least 3 months (*Class IIb, Level of evidence C*) (62). On the other hand, European groups have been stricter regarding allowance for high-intensity sports practice in patients with the potentially arrhythmogenic disease. This is reflected in a recent EHRA position paper, in which sports participation is only recommended provided lower risk factors are guaranteed (Table 3) (63). As an example, patients with arrhythmogenic cardiomyopathy and gene-mutation carriers should avoid competitive sports and high-intensity leisure physical activity as it may worsen ventricular function, trigger life-threatening ventricular arrhythmias and promote disease progression (64, 65). However, in young patients with mild disease, low-to-moderate exercise does not seem to be entirely detrimental and should not be deprived of the health benefits of such activity (65). In addition, in situations where there is an agreement to participate in all competitive sports, these more lenient recommendations are frequently accompanied by an exception including those in whom occurrence of syncope may be associated with serious harm or death (e.g., driving, climbing, and diving) (63).

**Table 3:** Disease-specific recommendation for sports practice in patients with potentially arrhythmogenic conditions.

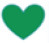 In patients with frequent **VBPs** or **NSVT**, if no indication of familial or structural underlying disease, all competitive and leisure-time sports activities are allowed (LoE C).
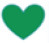 In case of ischaemia with or without **VT**, despite optimal medication and revascularization, only noncompetitive sports are allowed (LoE C).
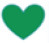 Athletes with idiopathic, monomorphic **VT**, without haemodynamic compromise during exercise, can resume competitive or leisure-time athletic disciplines^*^ (LoE C).
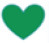 Athletes with idiopathic, monomorphic **VT** who have undergone successful VT ablation and are without any symptoms or other sign of recurrence during a 3-month follow-up period, can resume full competitive or leisure-time athletic activity (LoE C).
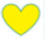 Athletes with idiopathic, monomorphic **VT** who choose to undergo drug treatment for suppression and are without any symptoms during a 3-month follow-up period, including exercise testing or EP study, may resume full competitive or leisure-time athletic activity (LoE C).
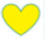 It is reasonable to allow all types of sports participation for asymptomatic athletes with an **LQT2** or **LQT3** mutation but QTc <470/480 ms, and who are on prophylactic beta-blocker therapy (LoE C).
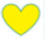 It is reasonable to allow individual sports at low to moderate intensity for asymptomatic athletes with an **LQT1** mutation but QTc <470/480 ms and who are on prophylactic beta-blocker therapy, but team sports and high-intensity sports are discouraged (LoE C).
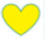 It is reasonable to allow light to moderate leisure sport activity to asymptomatic **SQTS** patients without family history of SCD (LoE C).
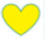 If there is no recurrent event during 3 months in symptomatic **BrS** patients after ICD implantation, leisure or competitive sports may be resumed based on shared decision-making (LoE C).
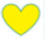 Asymptomatic **BrS** patients, asymptomatic mutation carriers, and asymptomatic athletes with only an inducible ECG pattern, may participate in all sports that are not associated with an increase in core temperature >39C° (LoE C).
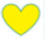 Patients with **CPVT**, under appropriate treatment, if stress-test shows absence of any type of ventricular ectopy/arrhythmia and if the patient is asymptomatic for a minimum of 3 months, low-intensity to moderate leisure-time sports may be considered, including those with an ICD (LoE C).
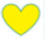 In individuals diagnosed with possible **AC** based on two minor criteria, sports eligibility should be considered on an individual basis after a comprehensive evaluation of the potential diagnosis (LoE C).
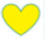 It seems reasonable that athletes with an unequivocal diagnosis of **DCM**, but mildly reduced LV systolic function (EF ≥ 40%) may selectively be allowed to participate in all competitive sports^*^, provided that specific low risk criteria^**^ are present (LoE C).
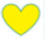 It seems reasonable that adult athletes with **HCM** may selectively be allowed to participate in all competitive sports^*^ if: (1) Mild clinical expressions of HCM (2) Low ESC risk score (3) Adult age (LoE C).

*EHRA position paper recommendations (63)*.

*
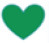
Indicates a “should do this” recommendation, based on at least one randomized trial, or is supported by strong observational evidence that it is beneficial and effective*.

*
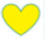
Indicates general agreement and/or scientific evidence favoring a “may do this” statement, based on randomized trials on a small number of patients or which is not widely applicable*.

*AC, arrhythmogenic cardiomyopathy; BrS, Brugada syndrome; CPVT, catecholaminergic polymorphic ventricular tachycardia; DCM, dilated cardiomyopathy; HCM, hypertrophic cardiomyopathy; LoE, level of evidence; LQT, long QT; NSVT, nonsustained ventricular tachycardia; SQTS, short QT syndrome; VBPs, ventricular premature beats; VT, ventricular tachycardia*.

* *Except those in which syncope may be associated with an enhanced risk for athlete or others (e.g., driving, climbing, diving)*.

** *(1) Asymptomatic, (2) Without prior history of unexplained syncope, and (3) without frequent/complex ventricular tachyarrhythmias on ambulatory ECG monitoring and exercise testing*.

### Efficacy and Safety of ICD in Athletes

Despite safety and efficacy concerns, many patients with ICDs continue regular sports practice, and some participate in competitions. The ICD Sports Safety Registry eased some of these concerns in competitive athletes (66). The investigators enrolled 440 athletes (10–60 years old) who were already engaged in organized competitive sports despite having an ICD. The most frequent diagnoses in this registry were LQTS (20%), HCM (17%), and arrhythmogenic right ventricular cardiomyopathy (13%). A 4-year follow-up study has shown that there were no cases of physical injury or failure to terminate arrhythmia despite participation in vigorous competitive sports (36). *Heidbuchel* et al. published a comparative analysis of these patients to 80 other patients with ICD who were participating in recreational moderate-to-high intensity sports, included in a parallel registry (67). They found similar safety and efficacy outcomes, as well as comparable freedom from 5- and 10-year probable or definite lead malfunction of 97 and 93%, respectively. On the contrary, in addition to the psychological benefits for an athlete, there may also be the potential cardiovascular benefit of continuing sports. A meta-analysis has also shown that in patients with heart failure and an ICD (mean age 54–66 years old, the majority with a history of myocardial infarction), exercise training was associated with significant improvement in cardiorespiratory fitness (68).

Although the agreement for sports participation should first be tailored to underlying disease of each patient (Table 3) (63), there are also general recommendations for patients who have an ICD. The AHA/ACC stated in 2015 that it is reasonable that patients with an ICD participate in sports with low dynamic and static components (e.g., golf, yoga, bowling) (69), as long as they are free from arrhythmic events requiring device therapy for 3 months (*Class IIa, Level of evidence C*) (58). In addition, participation in sports with higher intensity may be considered, taking into account the likelihood of appropriate or inappropriate shocks and device-related trauma (*Class IIb, Level of evidence C*) (58). In the European guidelines, shared decision-making relating to the continuation of intensive or competitive sports participation is recommended, taking into account the underlying disease, the psychological impact of shocks, the potential risk for third parties, and the fact that intensive sport will trigger more appropriate and inappropriate shocks (*Class IIa, Level of evidence C*) (63, 70). Empowerment of athletes with disorders with potential arrhythmic risk might have advantages such as allowing a more transparent and doctor–patient relationship, avoiding “doctor shopping” and acquiring more knowledge in “gray zone areas” (i.e., exercise in recipients of ICD) (71).

Participation in sports that involve collision (e.g., boxing or rugby) is not recommended because of the risk of damaging the device components, risk of hematoma formation, and subsequent pocket infection (70). For other team sports with some degree of physical contact (e.g., football, basketball, and baseball), a protective shield is recommended, although its effectiveness has never been proven (70). In addition, there are country-specific rules, such as in Italy where competitive sports eligibility for recipients of ICD can be granted in the following situations: (1) asymptomatic subjects; (2) no heart disease in which sport is contraindicated; (3) sports at low-moderate intensity; (4) sports without traumatic risk or with intrinsic risk; (5) sports in which the arm ipsilateral to the device is not repeatedly used; (6) at least 3 months after the last device intervention (72).

### Type and Implantation Technique of ICDs in Athletes

The choice of the type of ICD should primarily be based on the underlying disease and potential for arrhythmia. In general, a subcutaneous ICD should be considered in patients who pass screening test (i.e., large enough QRS and small T-waves) and have an indication for ICD when pacing therapy for bradycardia and cardiac resynchronization is not needed, or in whom sustained monomorphic ventricular tachycardia requiring antitachycardia pacing is not anticipated (*Class IIa, Level of Evidence C*) (57). There is no specific evidence supporting either transvenous or subcutaneous ICDs in athletes. In a propensity-matched case-control study of patients aged 35–40 years, the majority (60%) having a diagnosis of HCM and a mean ejection fraction of 58%, subcutaneous ICD was associated with a 70% relative risk reduction of device-related complications and inappropriate shocks, mainly because of higher rates of lead failures in the transvenous group (73). Despite a lower risk of complications at a mean of 31 months follow-up, subcutaneous ICDs were more expensive, even when accounting for the lower complication-related costs (73). Further comparisons of the safety and efficiency of both systems must be derived from other studies of populations (74, 75), such as the one studied by *Knops* et al. in the only randomized conrolled trial (RCT) published on this subject so far (the PRAETORIAN trial) (76). These were patients with a median age of 63 years, 69% with ischemic cardiomyopathy, and a median left ventricle ejection fraction of 30%, who indicated ICD but no indication for pacing (76). At a median follow-up of 49 months, subcutaneous ICD was noninferior to the transvenous ICD in terms of inappropriate shocks and device-related complications, as fewer lead-related complications were counterbalanced by more frequent pocket hematomas with the subcutaneous ICD (76). A meta-analysis of case–control studies derived similar results, and the reasons for inappropriate shocks differed between both groups: in the subcutaneous ICD, they were primarily due to oversensing (T-wave or noise), whereas in the transvenous ICD they were mostly due to supraventricular tachycardias (77). One can therefore argue that in athletes who do not need an antibradycardic device, a subcutaneous ICD should be preferred. Finally, the *ATLAS S-ICD* trial is an ongoing RCT (NCT02881255, estimated completion date—February 2022) that aims to study the benefit and risks of *Avoiding Transvenous Leads in Appropriate Subjects* who have either inherited arrhythmia syndrome, prior device removal for infection, need for hemodialysis, prior heart valve surgery or chronic obstructive pulmonary disease (78).

Some technical aspects should be considered during the implantation of an ICD in an athlete: namely (1) right-side approach in the case of left arm dominance; (2) submuscular placement of generator; and (3) axillary or cephalic venous access to prevent a subclavian crush (63, 70). In addition, device programming should contemplate adequate rate response pacing, higher detection zones, longer arrhythmia detection intervals, and proactive exclusion of myopotentials interference to prevent inappropriate shocks (63, 70). In a subanalysis of the ICD Sports Registry, detection rates > 200 bpm and detection intervals longer than nominal were associated with decreased risk of total and inappropriate shocks during competition or practice, and higher shock-free survival, respectively (79). To avoid inappropriate shocks during sports activity, a Holter ECG monitoring and exercise testing can be performed to evaluate maximal heart rate during effort and set a threshold for shock delivery at least 20 bpm above the maximal sinus rate observed (72). Finally, treating physicians should have a lower threshold for referring patients/athletes with ICD for ablation of atrial and ventricular arrhythmias that may be the cause of appropriate and inappropriate therapies (63).

## Conclusion

Sudden cardiac arrest in young athletes is a rare event, although accurate registries are needed to allow more accurate recording of SCA events to facilitate appropriate public health interventions. While some athletes with arrhythmic conditions may be allowed to continue sports practice, particularly in absence of structural heart disease or a channelopathy, some others should be disqualified from sports competition. In those who receive an ICD, special device and implantation choices may apply, and shared decision-making is recommended, taking into account the underlying disease, the psychological impact of shocks, and the type of sports. Nevertheless, athletes with ICDs may be excluded from competitions, depending upon country-specific and competition rules.

Cardiopulmonary resuscitation maneuvers are effective in preventing SCD and are responsible for an 8-times higher survival rates in sports-related SCA, compared with SCA that are not sports related. Initiatives to increase bystander delivery of CPR should be promoted by sports institutions and public health institutions, such as coordinated CPR training starting from school years, as part of the “Kids Save Lives” campaign. Although the distribution of AED in all sports clubs/venues might not be cost effective, further research and modeling into more cost-effective strategies are required but could include a quick and effective app-based mapping and location too to identify the nearest public access AED might help to save lives if an athlete or spectator collapses and requires resuscitation.

## Author Contributions

MK, FR, RP, CC, and MC conceived the idea for the work. MC and MK drafted the manuscript. RP, FR, CC, FD'A, and AC reviewed the manuscript and provided critical edits. All the authors approved the final manuscript and agreed to be accountable for the content of this study.

## Conflict of Interest

The authors declare that the research was conducted in the absence of any commercial or financial relationships that could be construed as a potential conflict of interest.

## Publisher's Note

All claims expressed in this article are solely those of the authors and do not necessarily represent those of their affiliated organizations, or those of the publisher, the editors and the reviewers. Any product that may be evaluated in this article, or claim that may be made by its manufacturer, is not guaranteed or endorsed by the publisher.
